# Extended antimicrobial treatment of bacterial vaginosis combined with human lactobacilli to find the best treatment and minimize the risk of relapses

**DOI:** 10.1186/1471-2334-11-223

**Published:** 2011-08-19

**Authors:** Per-Göran Larsson, Erik Brandsborg, Urban Forsum, Sonal Pendharkar, Kasper Krogh Andersen, Salmir Nasic, Lennart Hammarström, Harold Marcotte

**Affiliations:** 1 Department of Obstetrics and Gynaecology Kärnsjukhuset, Skaraborg hospital and University of Skövde, SE-541 85 Skövde, Sweden; 2 Bifodan AS, Bogbinderivej 6, DK-3390 Hundested, Denmark; 3 Clinical Microbiology, Department of Clinical and Experimental Medicine, Linköping University, SE-581 85 Linköping, Sweden; 4 Division of Clinical Immunology, Department of Laboratory Medicine, Karolinska Institutet at Karolinska University Hospital Huddinge, SE-141 86 Stockholm, Sweden; 5 FoU Skas, Skaraborg hospital, SE-541 85 Skövde, Sweden

## Abstract

**Background:**

The primary objective of this study was to investigate if extended antibiotic treatment against bacterial vaginosis (BV) together with adjuvant lactobacilli treatment could cure BV and, furthermore, to investigate factors that could cause relapse.

**Methods:**

In all, 63 consecutive women with bacterial vaginosis diagnosed by Amsel criteria were offered a much more aggressive treatment of BV than used in normal clinical practice with repeated antibiotic treatment with clindamycin and metronidazole together with vaginal gelatine capsules containing different strains of lactobacilli both newly characterised and a commercial one (10^9 ^freeze-dried bacteria per capsule). Oral clindamycin treatment was also given to the patient's sexual partner.

**Results:**

The cure rate was 74.6% after 6 months. The patients were then followed as long as possible or until a relapse. The cure rate was 65.1% at 12 months and 55.6% after 24 months. There was no significant difference in cure rate depending on which *Lactobacillus *strains were given to the women or if the women were colonised by lactobacilli. The most striking factor was a new sex partner during the follow up period where the Odds Ratio of having a relapse was 9.3 (2.8-31.2) if the patients had a new sex partner during the observation period.

**Conclusions:**

The study shows that aggressive treatment of the patient with antibiotics combined with specific *Lactobacillus *strain administration and partner treatment can provide long lasting cure. A striking result of our study is that change of partner is strongly associated with relapse of BV.

**Trial registration:**

ClinicalTrials.gov: NCT01245322

## Background

Bacterial vaginosis (BV) is a disease with unknown aetiology, characterised by loss or reduction of lactobacilli and increased numbers of anaerobes and Gram-negative rods [1]. It is one of the most frequent vaginal infections, and the most common symptom is malodours discharge.

The currently recommended treatment regimes for BV are oral or vaginal metronidazole or vaginal clindamycin [2]. Treatment efficacy is supposed to be high. In a Meta-analysis, the expected cure rate after one month was 70%-80% for metronidazole [3] and 82% for clindamycin [4]. However, in clinical practice these high efficacy rates are rarely observed. Based on our own review of published data, the efficacy is not more than 60% after 4 weeks [5]. Likewise in a follow up study, only 42% were BV free 12 months after therapy [6]. In order to reduce the number of patients who experienced relapse, Sobel et al treated women with recurrent BV with vaginal metronidazole gel for ten days, compared to the normal five days [7]. Women who responded to therapy were then treated with metronidazole gel twice weekly or placebo for 4 months, followed for another 3 months without therapy. By the end of 4 months, 75% were cured in the treatment group compared to 59% in the placebo group, but after 7 months only 49% in the treatment group versus 25% in the placebo group remained cured.

Lactic acid-producing bacilli are part of the normal bacterial microbiota of the vagina and have a physiological role in maintaining a low pH (<4.5), thus protecting against invasion by other micro-organisms [8]. Recent studies, using not only traditional phenotypic methods but also genotyping, have convincingly shown the most common vaginal lactobacilli species to be *L. crispatus, L. gasseri, L. iners *and *L. jensenii *[9] where *L. crispatus *is associated to a normal flora and *L. iners *more often to BV [10].

The treatment suggestion by Reid et al. [11] i.e. giving oral capsules of *L. reuteri *(formerly *L. fermentum*) and *L. casei var. rhamnosus*, was reported to be successful in 37% of the treated women as compared to 13% given placebo. In a study from Nigeria, the same strains of lactobacilli given orally after a one week course of oral metronidazole cured 88% of women with BV after 30 days compared to 45% in the metronidazole/placebo group [12]. These efficacy results have not been obtained by any other group.

We have previously performed a treatment study with addition of vaginal lactobacilli that did not improve the initial cure rate but the relapse rate was nearly 20% lower in the *Lactobacillus *treated group [13]. Because of the lower relapse rate noted in this study, we designed a new explorative study with novel, well characterised *Lactobacillus *strains of human vaginal origin [9].

The primary objectives of this study were to characterise lactobacilli of human vaginal origin and to investigate if more extended antibiotic treatment against BV, together with adjuvant lactobacilli treatment, could increase the cure rate and furthermore, to investigate factors that could influence relapse.

## Methods

### *Lactobacillus *strains

A total of 110 isolates (51 *L. crispatus*, 45 *L. gasseri *and 14 *L. jensenei*) previously isolated from the vaginal tract of 15 healthy Swedish women [9] were initially included in the study for characterisation and selection of strains for a colonisation study (Table 1). *Lactobacillus iners *isolates were discarded since these bacteria require special growth requirements, precluding future large scale production and commercial application. Lactobacilli were grown in MRS or Rogosa agar in anaerobiose or in MRS broth in a standing condition at 37°C.

**Table 1:** Typing of vaginal *Lactobacillus *isolates^a^

**Woman **^**b**^	Isolate	species	REP-PCR type	RAPD-PCR type	RAPD-PCR type
				RAPD3	RAPD4
C	4B1, 4R4, 4R5, 4R6, 4M6	*L. crispatus*	1	I	A
D	5B1, 5B2, 5B3, 5R4, 5R5, 5R6, 5M7, 5M8, 5M9	*L. gasseri*	2		
E	6B1, 6B2, 6B3, 6R4, 6R5, 6R6, 6M9	*L. gasseri*	3		
	6M7, 6M9	*L. gasseri*	4		
F	7B1, 7B2, 7B3, 7R4, 7R5, 7R6, 7M7, 7M8, 7M9	*L. gasseri*	5		
G	8B1, 8B2, 8B3, 8R4, 8R5, 8R6, 8M7, 8M8, 8M9	*L. crispatus*	6		
H	9R4, 9M7, 9M8, 9M9	*L. crispatus*	7		
J	12B1, 12B2, 12B3, 12R4, 12R5, 12R6, 12M7, 12M8, 12M9	*L. jenseneii*	8		
K	13B1, 13B2, 13B3, 13R4, 13R5, 13R6, 13M7, 13M8, 13M9	*L. crispatus*	9		
L	15R4, 15R5, 15R6, 15M7, 15M8, 15M9	*L. gasseri*	10		
M	16B1, 16R5, 16R6	*L. gasseri*	11		
	16B2	*L. crispatus*	12		
N	18B1, 18B2, 18B3, 18R4, 18R5, 18R6, 18M7, 18M8	*L. crispatus*	13		
P	20B31, 20B32, 20B33, 20R34, 20R35, 20R36, 20M39	*L. gasseri*	14		
	20M37, 20M38	*L. gasseri*	15		
Q	21R44, 21R45, 21R46, 21M47, 21M48, 21M49	*L. crispatus*	1	II	A
R	22B41, 22B42, 22R45, 22M48, 22M49	*L. jenseneii*	16		
S	23B31, 23B32, 23B33, 23R34, 23R35, 23R36, 23M37, 23M38, 23M39	*L. crispatus*	1	II	B

^a ^The different REP-PCR types were numbered from 1 to 16. Isolates from women 4, 21 and 23 were further differentiated by RAPD-PCR with primer 3 (type I and II) and RAPD 4 (type A and B).

^b ^Nomenclature according to Vásquez *et al *2002.

### Genotyping by REP-PCR and AP-PCR

The *Lactobacillus *strains were initially typed using PCR amplification on the bacterial repetitive extragenic palindromic DNA sequences (REP-PCR). Chromosomal DNA was isolated from lactobacilli grown in MRS using the DNAeasy kit (Qiagen GmbH, Hilden, Germany) according to the manufacturer protocol for gram positive bacteria. The primers REP1R-I (5'-IIIICGICGICATCIGGC-3') and REP2-I (5'-ICGICTTATCIGGCCTAC-3') were used based on the reproducibility, band intensity and discriminative power [14]. The amplification mixture contained 25 ng template DNA, 1 μM of each primer, 1 X Green GoTaq™ Flexi reaction buffer (Promega, Madison, Wisconsin, USA) and 200 μM of each dNTP, 3 mM MgCl_2_, and 2.5 U GoTaq™ DNA Polymerase for a total of 50 μl. Bacterial DNA was amplified by PCR using the same thermocycler (GeneAmp^® ^PCR System 9700, Applied Biosystem) and the cycling parameters described by Ventura and Zink [14]. Ten μl of the PCR product was electrophoresised on a 2% agarose gel containing 1 × TAE (Tris acetate-EDTA) and the DNA was visualised by UV transillumination using a Molecular Imager Gel Doc™ system (BioRad, Hercules, CA).

As the *L. crispatus *strains from women 4, 21 and 23 shared identical REP-PCR profiles, the strains were further differentiated using RAPD (Rapid amplified polymorphic DNA)-PCR with the primers RAPD3 (5'-ACGAGGCAC-3') (primer OPL-5, [15] and RAPD4 (5'-CCGCAGCCAA-3') (primer 1254, [16]). Furthermore, during the colonisation study, positive profile results obtained by REP-PCR were confirmed by two different RAPD PCR using two primers: RAPD3 and RAPD4 for *L. crispatus *strains; RAPD4 and RAPD6 (5'-TGGGCGTCAA-3') (primer OPL-2, [15]) for *L. gasseri *strains; and RAPD1 (5'-ATGTAACGCC-3') (primer P2, [17]) and RAPD4 for *L. rhamnosus*. Each primer was used at 0.5 μM employing the same PCR mixture as described for the REP-PCR. The cycling program was: initial denaturation at 94°C for 2 min; 35 cycles of denaturation at 94°C for 1 min; annealing at 35°C for 1 min; extension at 72°C for 2 min and a final extension at 72°C for 8 min. Ten *μ*l of PCR reaction was electrophoresised on a 2% agarose gel containing 1 X TAE (Tris acetate-EDTA).

### Characterisation of *Lactobacillus *strains

Eighteen *Lactobacillus *strains isolated from Swedish women and showing different REP- and RAPD PCR profiles were selected and tested for growth rate, aggregation, H_2_O_2 _production, hemolysis, antibiotic susceptibility and the presence of plasmids.

The growth rate was evaluated by inoculating a 10 ml MRS tube adjusted to an OD_600 _of 0.04 and measuring the time of growth from OD_600 _0.04 to OD 1.0 (late exponential phase). The value was used to calculate an approximate doubling time.

In order to measure aggregation, bacteria were grown until an OD_600 _1.0 in 10 ml MRS. The tubes were vortexed for 1 min and the aggregation was scored after 30 min in a standing position. The aggregation was scored as follows: no aggregation (0), presence of flakes (1), sedimented flakes but cloudy supernatant (2), sedimented flakes and clear supernatant (3).

H_2_O_2 _production by *Lactobacillus *strains was tested with MRS agar supplemented with 0.25 mg of tetramethylbenzidine (Sigma, St. Louis, Mo.) per ml and 0.01 mg of horseradish peroxidase (Sigma) per ml [18]. H_2_O_2 _production was visually scored as strongly positive (3), intermediate positive (2) weakly positive (1), or negative (0) according to the intensity of blue colour development.

The haemolytic activity was evaluated on Columbia blood agar plates containing 5% human or sheep blood following 48 h incubation at 37°C in aerobic, microaerobic (5% CO_2) _and anaerobic conditions. Two *Streptococcus pyogenes *clinical isolates (*S. pyogenes *PU1735 and *S. pyogenes *svt) obtained from Clinical Microbiology, Department of Clinical and Experimental Medicine clinical University of Linköping were used as positive controls.

The minimum inhibitory concentration (MICs) of ten antibiotics was determined by the E-test method according to the manufacturer's instruction for lactobacilli (AB Biodisk; Solna, Sweden). The breakpoints (susceptible/resistant) were determined in accordance with the Clinical and Laboratory Standards Institute guidelines for gram-positive microorganisms [19].

Plasmids were isolated from a 10-ml culture grown until OD_600 _0.8 using the following steps: treatment of cells with lysozyme (10 mg/ml) and mutanolysin (80 u/ml), cell lysis, neutralisation, extraction with phenol/chloroform/iso-amylalcohol and precipitation with ethanol. The nucleic acid pellet was washed once with 70% ethanol, dissolved in 100 μl of TE buffer (10 mM Tris-HCl, pH 8.0 and 1 mM EDTA) containing 100 μg/ml of RNase A. Ten μl was run on a 1% agarose gel and the presence of plasmids was visualized by UV transillumination.

### Lactobacilli used for treatment

Nine of the vaginal *Lactobacillus *strains isolated from healthy Swedish women and characterised in the present study were selected for the clinical trial. The lactobacilli were fermented, lyophilised, and dispensed as a mixture of three different strains (10^9^/capsule) in gelatin capsules according to GMP standard (Gruppo Clerici SACCO SS.r.l., Cadorago Italy together with Bifodan A/S, Hundested, Denmark). Other strains from commercial products, currently used for treatment of BV, were also tested for comparison. These strains include *L. gasseri *(Lba EB01-DSM 14869) and *L. rhamnosus *(Lbp PB01-DSM 14870) contained in the commercially available EcoVag^® ^vaginal capsules (10^8 ^CFU/capsules) (Bifodan A/S, Denmark). Both these *Lactobacillus *strains were initially isolated from healthy women in Norway. Capsules containing the EcoVag^® ^strains plus another one, *L. gasseri *DSM 15527, also isolated from healthy women at the same time and characterised by Bifodan. The second probiotic preparation used was LaciBios^® ^(oral capsules containing *Lactobacillus rhamnosus *GR-1 and *Lactobacillus reuteri *RC-14 (around 10^9^/capsule)). These *Lactobacillus *strains have been described in the studies by Reid and collaborators [11,20].

Women were divided in seven groups that received capsules containing two to three *Lactobacillus *strains as follows. Group 0: EcoVag^® ^capsule containing *L. gasseri *DSM 14869 and *L. rhamnosus *DSM 14870; Group 1: *L. crispatus *4R5, *L. gasseri *20M39, and *L. jenseneii *22B42; Group 2: *L. crispatus *23B33, *L. gasseri *6M9*, L. jenseneii *12B1; Group 3: *L. crispatus *21M49, *L. gasseri *6M9 and *L. crispatus *8R6; Group 4: *L. gasseri *DSM 14869, *L. rhamnosus *DSM 14870 and *L. gasseri *DSM 15527; Group 5: *L. gasseri *DSM 14869, *L. rhamnosus *DSM 14870, *L. gasseri *DSM 15527; Group 6: LaciBios^® ^femina containing *L. rhamnosus *GR-1 and *L. reuteri *RC-14. Group 0 to 4 received capsules intra vaginally while groups 5 and 6 took the capsules orally.

### Clinical Study

The study was conducted at an outpatient private gynaecological clinic in Drammen, Norway from January 2007 until January 2011. All women with symptomatic BV fulfilling the inclusion criteria were consecutively offered to participate in a prospective study of adjuvant lactobacilli given in addition to antibiotics. Women included were regularly menstruating women, 18 years or older, with normal gynaecological status, not pregnant or breast-feeding and without signs of other genital tract infections. Exclusion criteria were patients with hormonal IUD without regular menstruation; women infected with *Chlamydia trachomatis*, or *Trichomonas vaginalis*, or with a clinical candida infection.

#### Study sample

A total of 76 patients were included in the study. The mean age was 33.7 years with a range of 18-55 years. The diagnosis of BV was based on Amsel criteria. Of the 76 patients, 13 were lost to follow up so that the study at hand consists of 63 patients.

#### Clinical method

At inclusion, women had a routine gynaecological examination with a non-lubricated speculum and a vaginal ultrasound. A sample of vaginal secretion was analysed for vaginal pH using special pH strips (range 3.8-5.0). The diagnosis of BV was based on Amsel criteria [21], i.e. fulfilling at least 3 of 4 criteria; thin homogenous discharge, vaginal pH above 4.5, positive amine test, and presence of clue cell during microscopical investigation using a phase contrast microscope. A vaginal sample was taken and air-dried. At inclusion, vaginal samples for determining *Chlamydia trachomatis *infection were performed using strand-displacement amplification (CT amplified DNA assay; Becton-Dickinson) according to the local laboratory routine. Samples for *Neisseria gonorhoeae *culture were only taken when deemed clinically motivated.

#### Treatment

**Figure 1:**
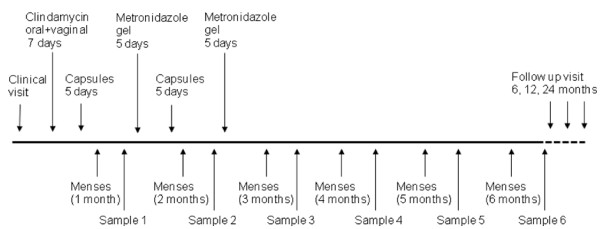
**Time schedule of the treatment and follow up**. Even if the patient had a relapse at the self taken samples (3-6 months) she did not receive treatment until the clinical visit that determined with Amsel criteria at the clinical visit after 6 months.

After signing the informed consent form, women were given a seven days course of daily 2% vaginal clindamycin cream (Dalacin vaginal cream 2%, Pfizer Norway Ltd) together with oral clindamycin 300 mg BID for 7 days (Dalacin 300 mg, Pfizer Norway Ltd). Oral clindamycin treatment was also given to the patient's sexual partner [22], however the patient's partner did not sign any informed consent forms. Directly after the clindamycin treatment, a new treatment with vaginal gelatine capsules containing 10^8^-10^9 ^freeze-dried lactobacilli for 5 days was started. If menstruation was to occur during treatment, it was suggested that the patient would wait until next menstrual cycle before she started the treatment. After the next menstruation, a new treatment with vaginal metronidazole for 5 days (Zidoval gel 75 g, Meda AS, Norway) was initiated followed by 5 more days with *Lactobacillus *instillation. After the second menstruation, the patients were given a new course of vaginal metronidazole gel (Figure 1). The evaluation of treatment efficacy was performed after the last treatment course.

#### Follow up

After every menstruation the patients took a self swabbed vaginal culture and a glass smear that was air dried and sent in a sealed envelope to the laboratory using the same method as in an earlier study [23]. The vaginal sample was collected after the bleeding had ceased and before the start of treatment.

The vaginal samples were sent by regular mail from Norway to the Clinical Microbiology laboratory, Linköpings University, Sweden where it was cultured. All recovered lactobacilli were identified to the genus level *Lactobacillus *by Gram staining, colony morphology and negative catalase test and were subsequently frozen at -70°C and transported to the Clinical Immunology laboratory at the Karolinska hospital in Huddinge where additionnal characterisation was performed. All recovered lactobacilli isolates were again cultured and identified to the species level by amplifications of 16S rDNA regions using species-specific primers. Identification to the strain level was carried out using REP-PCR and RAPD PCR (as described above) to test if they were the same strains as those provided in the lactobacilli-containing capsules. These tests were performed blindly, without knowledge of the treatment the women had received.

Every woman was followed up by a phone call from the investigation nurse and asked about treatment complications and concomitant medication. If the patient had failed to send a sample, it was recorded as missing but she was reminded and asked to send in a new sample after the next menstruation. Each patient collected a total of 6 vaginal samples.

After six menstrual cycles, women were scheduled for a follow up visit to the clinic. Normally the vaginal samples were taken once every 28^th ^day and the follow up time of 6 menstrual cycles would be 5 and half month but for some women 6 menstruation cycles could take longer than 6 months, thus, we report this as a 6-month follow up.

The women were then followed up to 12 months and they were offered a new visit at 24 months, or before, in the case of symptomatic relapse.

#### Microscopical investigation

Each vaginal sample was rehydrated with normal saline and investigated under a phase contrast microscope with 400 times magnification and with a microscopic area of 0.016 mm^2^. The approximate numbers of bacteria were recorded as well as the presence of clue cells together with the number of vaginal leucocytes. Each slide was classified according to Hay/Ison classification [24,25] to either normal, intermediate or BV. All microscopic evaluations were performed after that the women had been on clinical visits.

#### Definition of cure

To be considered cured, the women had to have none of Amsels critera fulfilled at the clinical visit after 6-9 months. All women with clinical relapse (fulfilment of Amsel criteria) at the clinical visit were regarded as treatment failures and were re-treated with clindamycin and metronidazole. Treatment efficacy is reported both as cure after 6, 12 and 24 months, and as time to relapse illustrated with survival analysis with Life table analysis. Some women had treatment failure already at the third month as seen on the self taken sample. These were considered as treatment failures if the Hay/Ison score was 3, a Hay/Ison score of 2 was designated as improved and a score of 1 as cure. A Hay/Ison score of 0 and 4 were regarded as equal to a score of 1 during the follow up periods. As all microscopic investigations were performed after that the women had been on a clinic visit and re-treatment was not initiated on the microscopic results.

#### Study design

The study was performed as a consecutive treatment, open label, follow-up study.

#### Statistical methods

Not all patients collected a vaginal smear every month and in case of missing data during the first follow up month, the procedure "last observation carried forward" (LOCF) was used. "Time from treatment to relapse" has been calculated in months and was used to explore differences in time to relapse between groups by survival analysis with Kaplan Meier procedure. A logistic regression analysis was performed and an Odds ratio (OR) with 95% confidence interval (CI) was calculated. All comparisons both between and within groups was performed two-tailed with a significance level of 5%. All the analyses in this study were performed in PASW (SPSS) v.18 and in accordance with the principal of intention to treat (ITT).

#### Ethical approval

The study was approved by the southeast regional Ethics Committee in Oslo, Norway. To recruit women with BV, we advertised in local newspapers asking for women suffering from malodorous discharge.

## Results

### Typing and characterisation of vaginal lactobacilli

Analysis of the lactobacilli previously isolated from healthy women showed that only one *Lactobacillus *species of the same REP-PCR type was generally found among the isolates from each woman. The only exceptions were the women E and P who both carried *L. gasseri *isolates with two different REP-PCR profiles and woman M who carried two species (*L. gasseri *and *L. crispatus*) (Table 1). Strains from different women showed different REP-PCR profiles apart from the *L. crispatus *strains from woman C, Q and S. These *L. crispatus *strains could further be differentiated by performing two RAPD-PCR with the primers RAPD3 and RAPD4 (Table 1). A total of 18 different genotypes were identified by using a combination of REP-PCR and RAPD-PCR.

Eighteen *Lactobacillus *strains with different PCR types were selected for further characterisation and were scored according to properties that would promote their colonisation (auto aggregation) and antimicrobial capacity (H_2_O_2 _production), guarantee their safety for human use (abscence of plasmid, absence of hemolysis and no antibiotic resistance) and possibility for large scale production (growth rate) (Table 2). Four strains containing plasmids (5M8, 13B2, 15M7, 16B2) and one strain with ambiguous blood hemolysis results (18R5) were discarded. Although the hemolysis could not be classified as β-hemolysis, it was decided to not pursue further studies with this strain for potential safety reasons. None of the strains were resistant to ampicillin, cefuroxime, imipenem, gentamicin, erythromycin, chloramphenicol, tetracycline, or rifampicin. All the tested lactobacilli were resistant to metronidazole and several of the strains (10 of 18 strains) were resistant to cefoxitin. High level of resistance to these antibiotics has been previously reported and is considered as natural resistance in lactobacilli [26].

**Table 2:** Characterisation of vaginal *Lactobacillus *strains

PCR type strain	Species	**Doubling time**^**a**^	**Autoaggregation**^**b**^	**H**_**2**_**O**_**2**_^**c**^	Hemolysis	**Plasmid**^**e**^	**Antibiotic resistance**^**f**^	**Total score**^**g**^
23B33	*L. crispatus*	92 (1)	3	3	α	-	MZH, FX	7*
21M49	*L. crispatus*	91 (1)	3	2	α	-	MZH	6*
8R6	*L. crispatus*	83 (2)	3	0	α	-	MZH	5*
4R5	*L. crispatus*	109 (1)	3	0	α	-	MZH	4*
9M9	*L. crispatus*	102 (1)	1	2	α	-	MZH	4
13B2	*L. crispatus*	96 (1)	2	1	α	+	MZH	0
16B2	*L. crispatus*	113 (1)	2	1	α	+	MZH	0
18R5	*L. crispatus*	95 (1)	2	3	? ^d^	-	MZH, FX	0
6M9	*L. gasseri*	69 (2)	3	1	α	-	MZH, FX	6*
6M8	*L. gasseri*	71 (2)	3	0	α	-	MZH, FX	5*
20M39	*L. gasseri*	68 (2)	0	1	α	-	MZH, FX	3*
20M37	*L. gasseri*	71 (2)	0	1	α	-	MZH, FX	3
16B1	*L. gasseri*	122 (0)	1	1	α	-	MZH, FX	2
7B3	*L. gasseri*	183 (0)	0	1	α	-	MZH	1
5M8	*L. gasseri*	77 (2)	3	1	α	+	MZH, FX	0
15M7	*L. gasseri*	68 (2)	1	0	α	+	MZH, FX	0
22B42	*L. jensenii*	85 (2)	3	2	α	-	MZH, FX	7*
12B1	*L. jensenii*	71 (2)	1	2	α	-	MZH, FX	5*

^a ^Doubling time: more than 120 min (score 0), between 90-120 min (score 1), between 60-90 min (score 2)

^b^Autoaggregation: no aggregation (score 0), presence of flakes (score 1), sedimented flakes but cloudy supernatant (score 2), sedimented flakes and clear supernatant (score 3).

^c ^H_2_O_2 _production: strongly positive (score 3), intermediate positive (score 2) weakly positive (score 1), or negative (score 0) according to the intensity of blue colour development.

^d ^Slight lysis of human blood cells were observed for 18R5 when grown in anaerobiosis.

^e ^Presence (+) or absence (-) of plasmid

^f ^Antibiotics to which the *Lactobacillus *strains are resistant among the 10 tested. Metronidazole (MHZ), cefoxitin (FX).

^g ^Total score for a *Lactobacillus *strain is the sum of scores for doubling time, auto-aggregation and hydrogen peroxide production (max. value 8). Strains showing slight hemolysis (18R5) and presence of plasmid (5M8, 13B2, 15M7, 16B2) were scored 0.

*Strains with the highest score selected for the clinical trial.

Nine *Lactobacillus *strains of different species and presenting the highest scores were selected for the clinical trial: three *L. gasseri *(6M8, 6M9, 20M39), four *L. crispatus *(4R5, 8R6, 21M49, 23B33) and two *L. jenseneii *(12B1, 22B42) strains (Table 3).

**Table 3:** Demographic and clinical characteristics in the study population at enrolment (n = 63)

**Groups **^**a**^	0	1	2	3	4	5	6	Total
	
	EcoVag^®^	*Lc *4R5	*Lc *23B33	*Lc *21M49	*Lg*14869	*Lg *14869	LaciBios^®^	
	*Lg *14869	*Lg *20M39	*Lg *6M9	*Lg *6M9	*Lr *14870	*Lr *14870	*Lr *GR-1	
	*Lr *14870	*Lj *22B42	*Lj *12B1	*Lc *8R6	*Lg *15527	*Lg *15527	*Lr *RC-14	
	
	Vaginal	Vaginal	Vaginal	Vaginal	Vaginal	Oral	Oral	
	
	n = 9	n = 12	n = 10	n = 10	n = 9	n = 4	n = 9	n = 63
Mean age (range)	34.2 (19-55)	36.6 (26-48)	35.3 (25-45)	32.7 (25-39)	33.3 (21-46)	33.0 (25-38)	34.7 (21-45)	34.2 (19-55)
Patients with history of BV (no.)	3	6	5	5	3	0	1	23 (36.5%)
Patients with *Mobiluncus *on microscopy (no.)	4	6	5	3	4	2	2	26 (41.3%)
Length of symptoms of BV (months, mean and range)^c^	10.7 (1-24)	10.7 (1-48)	14.1 (1-60)	7.9 (1-48)	18.3 (2-60)	3.3 (2-6)	9.5 (1-24)	11.3 (1-60)
Patients with new relation (no.)	8	6 (2 divorced)	8 (1 divorced)	6 (2 divorced)	6	3 (1 divorced)	4	36 (57.1%)
New relation before symptoms appear (no.)	8	6	8	6	6	3	3	35 (55.6%)
Length of symptoms of BV among women with new relation (months, mean and range)	10.5(1-24)	6.5 (1-12)	14.4 (1-60)	4.5 (2-11)	11.3 (2-46)	3.3 (2-6)	11.2 (1-24)	8.2 (1-46)

^a ^Each group are given capsules with a mix of two or three *Lactobacillus *strains: *Lg*, *L. gasseri*; *Lr*, *L. rhamnosus*; Lc, *L. crispatus*; Lj, *L. jenseneii*

^b ^Capsules contain the two strains from EcoVag^® ^plus an additional strain, *L. gasseri *DSM 15527, belonging to Bifodan A/S

^c ^One patient in group 3, 5, 6 and two patients in group 4 could not give any information since how long time she had symptoms of BV.

### Clinical treatment with antibiotics and lactobacilli

#### Demographic and behavioural characteristics

Of the 76 patients enrolled, 13 were lost during the follow up. Considering that 14 women got pregnant during this period, the 17% drop out rate is comparable to other studies [7,6]. The demographic and behavioural characteristics of the 63 participants that completed the study are shown in Table 3. All the women included were Caucasians. There was no difference in the above characteristics between the women in the different treatment groups.

Twenty-three women (36.5%) had been treated for BV previously with a mean time of 3.8 years before starting the study. There was no data on how many times the women had been previously treated for BV. The median time that the symptoms of BV had started was 5 months before the start of the study with a range of 1-60 months and the average time was 10.8 months.

Women reported that they had met a new partner in 36 cases (57.1%) and 6 women (9.5%) had recently been divorced and only 21 (33.3%) had the same sexual partner. The median time for the new relationship was 6 months with a range of 1-48 months and the mean time was 12 months.

Among the 36 women that had a new partner, one woman reported that she had symptoms of BV before she met her new partner. Among the other 35 patients, it is then possible to calculate an "incubation time". That is the difference between the time that the patient starts her new relationship and the beginning of symptoms of BV. The median time was 2 months with a range of 1-22 months and a mean time of 3.8 months.

#### Colonisation

Colonisation with any given lactobacilli was observed in 17 out of the 43 patients that received intravaginal lactobacilli (Table 4). Four women were not included in the analysis of colonisation as they did not provide enough samples. The women receiving EcoVag^® ^(Group 0) were colonised by *L. rhamnosus *14870 in 6 out of 8 cases (75%). The strain persisted more than 2 months in three patients, i.e. about 2 weeks after stopping its administration, and until Month 3 and 5 in two other patients (one and three months after stopping the treatment respectively). Of the women receiving the characterised lactobacilli isolated from Swedish women (Group 1 to 3), the *L. crispatus *strains were the ones showing the best colonisation (Table 4). Although colonising a lower proportion of women (around 33%), *L. crispatus *4R5 (Group 1), *L. crispatus *23B33 (Group 2), and *L. crispatus *8R6 (Group 3) persisted until Month 6 in 7 out of 9 women colonised by one of these strains i.e. 4 months after stopping the treatment. Of the women receiving a mixture of *L. rhamnosus *14870, *L. gasseri *14869 and *L. gasseri *DSM 15527, only two were colonised either by *L. rhamnosus *14870 or *L. gasseri *14869. None of the women that took the *Lactobacillus *preparation orally (Group 5 and 6) got colonised.

**Table 4:** Colonisation of the vaginal tract by administered *Lactobacillus*

Group	Treatment	**Number of women included per group **^**a**^	Number of women colonised by each strain (%)	
0	Vaginal	8	Any strain	6 (75%)
			*L. gasseri *Lba EB01-DSM 14869	0 (0%)
			*L. rhamnosus *Lbp PB01-DSM 14870	6 (75%)
1	Vaginal	9	Any strain	2 (22%)
			*L. crispatus *4R5	2 (22%)
			*L. gasseri *20M39	1 (11%)
			*L. jenseneii *22B42	0 (0%)
2	Vaginal	9	Any strain	4 (44%) ^b^
			*L. crispatus *23B33	4 (44%)
			*L. gasseri *6M9	1(11%)
			*L. jenseneii *12B1	0 (0%)
3	Vaginal	9	Any strain	3 (33%) ^b^
			*L. crispatus *8R6	3 (33%)
			*L. gasseri *6M9	1 (11%)
			*L. crispatus *21M49	0 (0%)
4	Vaginal	8	Any strain	2 (25%) ^b^
			*L. gasseri *(Lba EB01-DSM 14869)	2 (25%)
			*L. rhamnosus *(Lbp PB01-DSM 14870)	1 (13%)
			*L. gasseri *DSM 15527^a^	0 (0%)
5	Oral	4	Any strain	0 (0%)
			*L. gasseri *(Lba EB01-DSM 14869)	0 (0%)
			*L. rhamnosus *(Lbp PB01-DSM 14870)	0 (0%)
			*L. gasseri *DSM 15527^a^	0 (0%)
6	Oral	9	Any strain	0 (0%)
			*L. rhamnosus *GR-1	0 (0%)
			*L. reuteri *RC-14	0 (0%)

^a ^Only women that sent samples regularly were included in the caculation.

^b ^Some women were colonise by two strains.

#### Clinical outcome

Of the 63 patients that completed the study, 28 had relapses after 24 months. The cure rate was 74.6% after 6 months, 65.1% after 12 months and 55.6% after 24 months (Table 5).

**Table 5:** Clinical outcome

**Groups **^**a**^	0	1	2	3	4	5	6	Total
	
	EcoVag^®^	*Lc *4R5	*Lc *23B33	*Lc *21M49	*Lg*14869,	*Lg *14869	LaciBios^®^	
	*Lg *14869	*Lg *20M39	*Lg *6M9	*Lg *6M9	*Lr *14870,	*Lr *14870	*Lr *GR-1	
	*Lr *14870	*Lj *22B42	*Lj *12B1	*Lc *8R6	*Lg *15527	*Lg *15527	*Lr *RC-14	
	
	Vaginal	Vaginal	Vaginal	Vaginal	Vaginal	Oral	Oral	
	
	n = 9	n = 12	n = 10	n = 10	n = 9	n = 4	n = 9	n = 63
6-month cure (no. and percentage)	8 (89%)	9 (75%)	8 (80%)	6 (60%)	6 (67%)	2 (50%)	8 (89%)	47 (74.6%)
12-month cure (no. and percentage)	7 (78%)	7 (58%)	6 (60%)	7 (70%)	6 (67%)	2 (50%)	6 (67%)	41 (65.1%)
24-month cure (no. and percentage)	5 (56%)	4 (33%)	6 (60%)	6 (60%)	6 (67%)	2 (50%)	6 (67%)	35 (55.6%)
Number of patients with new relation during follow up (no. and percentage of all)	2 (22%)	4 (33%)	4 (40%)	4 (40%)	2 (22%)	3 (75%)	3 (33%)	22 (34.9%)

^a ^Each group are given capsules with a mix of two or three *Lactobacillus *strains: *Lg*, *L. gasseri*; *Lr*, *L. rhamnosus*; Lc, *L. crispatus*; Lj, *L. jenseneii*

**Figure 2:**
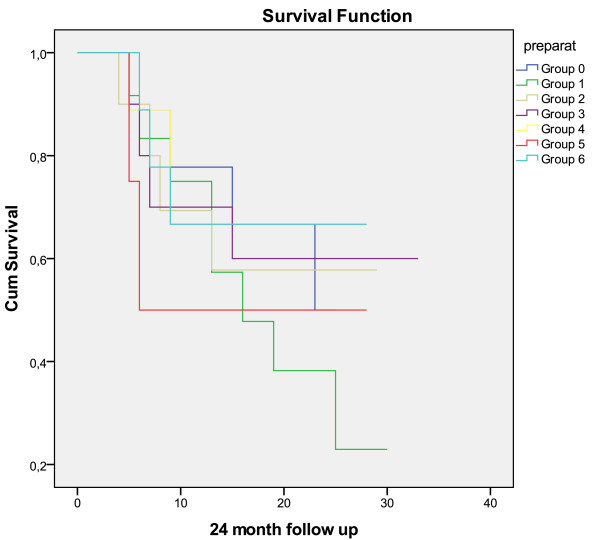
**Survival analysis with Life table showing the difference in cure rate between vaginal and oral administration of different *Lactobacillus *preparations**. No statistical difference is seen. Group 0: EcoVag^® ^capsule containing *L. gasseri *DSM 14869 and *L. rhamnosus *DSM 14870; Group 1: *L. crispatus *4R5, *L. gasseri *20M39, and *L. jenseneii *22B42; Group 2: *L. crispatus *23B33, *L. gasseri *6M9*, L. jenseneii *12B1; Group 3: *L. crispatus *21M49, *L. gasseri *6M9 and *L. crispatus *8R6; Group 4: *L. gasseri *DSM 14869, *L. rhamnosus *DSM 14870 and *L. gasseri *DSM 15527; Group 5: Oral (the same group as the vaginal group 4) *L. gasseri *DSM 14869, *L. rhamnosus *DSM 14870, *L. gasseri *DSM 15527; Group 6: oral LaciBios^® ^femina containing *L. rhamnosus *GR-1 and *L. reuteri *RC-14.

There was no significant difference in cure rate depending on which lactobacilli were given to the women (Figure 2). Furthermore, no correlation was found between colonisation with administered lactobacilli and the cure rate either at 6, 12 or 24 months after the start of the study.

**Figure 3:**
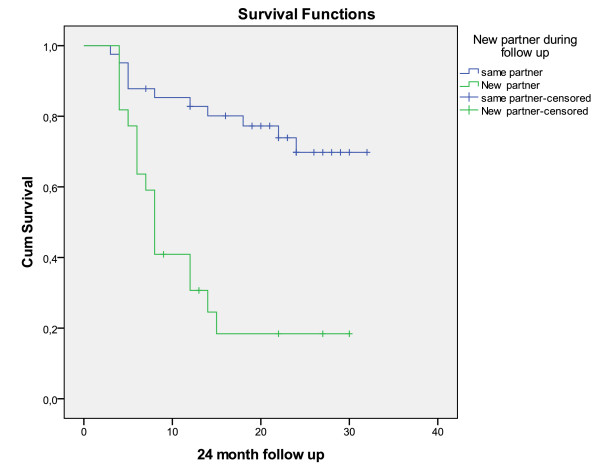
**Survival analysis with Kaplan-Meier showing that there were significantly more relapses in patients who report a new sexual partner during follow up**. Log rank < 0.001. Patients that are censored means that they have not completed the 24-month follow up. For some women the 24-month follow up is not done until 30 month. That is why some patients are reported as 30 month.

All patients' sexual partners, except two, accepted the oral clindamycin treatment. In one case, the couple had separated and partner treatment was not indicated. After a year, they started the same relationship again and this case was calculated as a new sexual relationship. In the second case, the partner refused to take any medicine. The patient had a microscopic relapse already at 4 months and a clinical relapse at the 6-month visit. This case is reported as having the same sexual partner. Two women reported the same male sexual partner but a new female sexual partner and are thus reported as having a new sexual partner. During the follow up time, a total of twenty-two patients had changed to new sexual partner. Of the 22 patients with a new partner, only 5 had no relapse (cure of 22.7%) compared to 30 of the 41 (73.2%) that did not change partner, OR = 9.3 (95% CI: 2.8-31.2). Survival analysis (Figure 3) showed that women who reported a new sexual partner during follow-up had significantly shorter time to relapse with a Log Rank test (p < 0.001).

Also a multiple logistic regression show that changed to new sexual partner during follow up was significantly associated to have a relapse but not age, earlier treatment of BV, presence of *Mobiluncus *before entering the study, a new relation before entering the study, lactobacilli given or whether or not the patient were colonised with any lactobacilli (Table 6). Of the 28 women with relapse, 21 had one relapse and five had two relapes. Two women had 4 and 5 relapses during the follow up period.

**Table 6:** Multiple logistic regression model where relapse within 24 months was outcome variable.

Variable	OR	95% confidence	interval
Age	1.1	1.0	1.2
*Lactobacillus *mix used for treatment	0.6	0.4	1.0
*Mobiluncus *at entry of the study	0.7	0.2	3.1
Pregnancy or major operation	1.3	0.6	2.8
New sexual partner during follow up	52.4	5.3	516.7
BV before study	1.3	0.3	5.8
New partner before study	0.4	0.1	1.2
Colonisation by lactobacilli	0.2	0.0	1.4

During the study, only one severe adverse event occurred, a case of diarrhoea in group 1 that caused the patient to stop treatment. There were no more candida infections than expected normally (10%).

## Discussion

The purpose of this study was to optimise the treatment of BV using extended antibiotics administration combined with administration of lactobacilli. By giving the patient a more aggressive treatment, we could obtain a 6-month cure rate of 74.6%, a 12-month cure rate of 65.1% and a 24-month cure rate of 55.6%. Interestingly, we could not find any difference in cure rate whether or not it was a recurrent BV. These findings are surprising as it has always been suggested in the literature that recurrent BV is more difficult to treat [7,27].

There are many treatment studies of BV that report cure rates of 90% but such a high cure rate is not seen in clinical practice [5]. As diagnostic tests for BV are subjective, one can question a cure higher than 70% after one month. From our own meta-analysis, a 4-week cure rate is never better than 60% [5]. Most studies report a 2 week or 4 week cure rate and there are very few studies that report a longer follow up than 4 weeks. As our treatment lasted 2 months, our 6-month cure rate corresponds to 4 months, or 4 menstruations, after the cessation of treatment. Our study could thus be compared to the Sobel study [7] where 88% of the women who clinically responded to a ten days treatment with 0.75% metronidazole gel were randomly assigned to metronidazole or placebo twice weekly for 4 months. Three months after cessation of treatment, the cure rate was 49% in the treatment group and 25% in the placebo group. But as only 88% were initially cured the comparable cure rate would be 43% in the treatment group, and this cure rate should be compared to our cure rate of 74.6%. In another follow up study from Australia, only 31% had a Nugent score of less than 3, 12 months after therapy with one week of oral metronidazole [6]. In comparison to these studies, our 6-month cure rate of nearly 75% is markedly superior.

Recurrence rates for BV beyond 12 months have been reported in very few studies. In one of them, non-pregnant women were treated with oral metronidazole for 10 days. The patients were treated until they were cured at a follow up visit and for some patients, the metronidazole treatment was repeated up to 3 times before all patients were cured. Outpatients' attendance was retrospectively reviewed, and follow-up at 6 years revealed a cumulative recurrence rate of BV of 53% where 73% of the recurrences had occurred within 12 months, i.e. a 12-month cure rate of 63% but starting from only cured patients [28].

Marcone *et al *have carried out adjuvant treatment with lactobacilli following treatment with oral metronidazole for 7 days [29]. The cure rate at 6 and 12 months was higher (>80%) than in the present study. However, the cure rate in women receiving the traditional treatment with metronidazole only was also much higher (around 70%) than the one previously reported in other studies (less than 36%) (Table 7) [6,7]. The definition of cure in their study was not based on Nugent or Hay/Isons scoring and only women who use natural methods of contraception were included, making it difficult to compare with other studies. The women selected in their study were probably engaged in a more stable relationship and less prone to change of partners and relapses. In a subsequent study by Marcone *et al*, long-term treatment with lactobacilli for 6 months resulted in a cure rate of 91% 6 months after stopping the treatment [30]. The study could be criticized for the same reason as the earlier study [29] but it could also be that the two courses of lactobacilli for 5 days used in the present study is a too short treatment and that a long-term treatment is necessary to obtain a stable biofilm in the vagina.

**Table 7:** Study results of suppressive treatment of BV.

		Cure rate after			
**Study**	**Treatment**	**>1 month**	**3 months**	**6 months**	**12 months**

Sobel 1993 [50]	Oral metronidazole 7 days		25%		
	Topical clindamycin 7 days		38%		
Boris 1997* [28]	Oral metronidazole 10 days (for some 3 times)				63%
Eriksson 2005** [23]	Topical clindamycin 3 days + lactobacilli tampons	57%			
	Topical clindamycin 3 days + placebo tampons	60%			
Sobel 2006 [7]	10 days vaginal metronidazole + twice weekly for 4 months		43%		
	10 days vaginal metronidazole + placebo		19%		
Bradshaw 2006 [6]	Oral metronidazole 7 days		46%	36%	31%
Schwebke 2007 [51]	Metronidazole gel twice weekly for 6 months			33%	
Marcone 2008 [29]	Oral metronidazole 7 days		71%	67%	
	Oral metronidazole 7 days + *Lactobacillus rhamnosus *for 2 months		88%	83%	
Larsson 2008 [13]	Topical clindamycin 7 days + lactobacilli			53%	
	Topical clindamycin 7 days + placebo			39%	
Reichman 2009 [52]	Triple treatment with oral nitroimidazole		65%	50%	
	21 days boric acid intravaginal and				
	Metronidazole gel 20 weeks (6 month treatment)				
Ehrnström 2010 [37]	Vaginal clindamycin + 5 types of lactobacilli			50%	
Marcone 2010 [30]	Oral metronidazole 7 days			74%	69%
	Oral metronidazole 7 days + *Lactobacillus rhamnosus *for 6 months			96%	91%
This study	Oral and vaginal clindamycin + vaginal metronidazole + lactobacilli			74.6%	65.1%
*Only cured patients were followed and some were treated 3 times.					

**minimum 2 menstruations periods.

If we exclude studies that report a 2 or 4-week cure rate, the treatment proposed in the present study describes one of the best cure rates that has ever been reported. Our treatment suggestion is extremely aggressive and one must ask if this aggressive treatment is justified. For many patients, however, BV is a burden that causes a high morbidity and it takes a long time and much effort in order to be cured. Often the women are treated with many courses of antibiotics so in the end, the difference to our aggressive treatment is that we give all antibiotics at one time instead of spreading it out over a longer time. The risk associated with aggressive antibiotic treatment is subsequent infection with *Clostridium difficile*. Clindamycin is a drug that is often associated with a risk for colitis but other antibiotics such as penicillin, ampicillin, cephalosporin and fluoroquinolones can all precipitate this disorder when used long term. As we only gave one course of treatment for 7 days with clindamycin, followed by two vaginal treatments with metronidazole, we think that our treatment regimen does not carry a high risk of acquiring *Clostridium difficile *infection [31] and during our study, only one patient had to withdraw treatment because of diarrhoea. Recurrent BV is troublesome and there have been only a few published studies on how to handle recurrent BV [6,27,32-36] with different suggestions as how to handle this problem. Women are often given treatment similar to suppressive treatment of chronic vulvo-vaginal candida infection [37].

It is not easy to perform a study in young women that continues for a period of 24 month. During this follow up, 14 women became pregnant and 5 underwent major surgery such as hysterectomy (data not shown). This could certainly influence the results of our 24 month cure rate at follow up and this has to be taken into consideration. As some patients still come to our clinic we have registered 4 more relapses (2 because of a new relationship) but these have been after more than 30 months after the first treatment. As we give the same aggressive treatment in the case of a relapse, only 2 women had more than 2 relapses. In at least one case, she had a sexual partner that was not monogamous.

Several clinical trials have been performed to investigate whether specific strains of lactobacilli, administered either orally or intra-vaginally, in combination with antibiotics or not, are able to colonise the vaginas of women with bacterial vaginosis and to improve symptoms and/or signs of bacterial vaginosis [12,29,38,39]. Our previous study with adjuvant EcoVag^® ^vaginal capsule treatment showed that lactobacilli could increase the cure rate at 6 months by nearly 20% (from 46% to 65%). However, only the women who responded to treatment were included in that study [13]. If we consider the women who were re-treated already after the first month, the cure rate drops to 53% in women treated with lactobacilli compared to 39% in the placebo treated group. Since a placebo group was included in the previous study, we did not include this group in the present one for ethical reasons. However, the cure rate obtained with vaginal administration of the new preparation tested was similar to the results obtained with EcoVag^® ^capsules. Among the new characterised *Lactobacillus *strains tested, only the *L. crispatus *strains were able to colonise. The strains colonised a lower number of individuals than *L. rhamnosus *DSM 16870 from the Ecovag^® ^capsule (30% vs 75%) but for a longer period (up to four months after stopping the treament). In comparison, the well studied probiotic strains *L. crispatus *CTV-05, *L. rhamnosus *GR-1 and *L. fermentum *RC-14 have been reported to colonise between 20 and 40% of the non antibiotic treated women 4-weeks post intravaginal administration [40-42]. In a more recent study, vaginal capsules containing a mixture of *L. gasseri *LN40, *L. fermentum *LN99, *L. casei *subsp. *rhamnosus *LN113 and *P. acidilactici *LN23 were administered for five days to women after conventional treatment with clindamycin. Following the first menstruation after *Lactobacillus *administration, 53% of the women were colonised by any of the five strains but only 26% after the second menstruation. In accordance with our results, no correlation was found between colonisation and treatment result of BV. The difference in colonisation of the vaginal tract by the probiotic lactobacilli might depend on factors such as vaginal intercourse and the microflora at enrollement [40].

Oral treatment with *Lactobacillus rhamnosus *(GR-1) and *Lactobacillus reuteri *(RC-14) have been shown to be extremely effective over a four week follow up period with a 90% cure [12]. In our study, however, oral treatment with the same lactobacilli did not performed as well over a long follow up period (67% cure).

Verstraelen et al. have reviewed the epidemiology of BV in relation to sexual behavior and concluded that BV in some acts as an STD or SED (sexually enhanced disease) and with frequency of intercourse being the critical factor [43]. A striking result of our study is that change of partner is strongly associated with BV, giving strong support to the notion of BV as a STD associated condition with consequence for treatment/partner treatment. In the study of Bradshaw [6], the authors found that having a regular sex partner was associated with a higher recurrence rate whereas having a new sex partner was associated with a lower recurrence. They suggested that women treated for BV are reinfected by their regular partner but not by new partners who would then be less likely to carry the causative agent for BV. The discrepancies between these two studies could be explained by the fact that we treated the partners with clindamycin. However, according to published studies, there is no evidence that partner treatment is efficacious [44]. The most quoted studies are the Scandinavian studies by Vejtorp [45] and Moi [46] where the authors used 2 grams of metronidazole at day 1 and 3 given to the female and to the male either as placebo or metronidazole in the same dose. They reported cure rates of 75-77% regardless of whether the partner was treated or not. Both studies were carried out at a STD clinic and none of the studies controlled for a new partner during the follow up period. This treatment regimen of metronidazole (2 grams, day 1 and 3) has been used worldwide but in fact this treatment has never been evaluated in a placebo-controlled study. Three other studies have reported a tendency towards a better cure rate if the partner was treated [47-49]. The only study that used clindamycin reported a 10%, non-significant, increase in cure rate after treating the partner with oral clindamycin [22]. So as a conclusion from our study and from the critical review of the literature we suggest that partner treatment should be used more often.

It is surprising that women tend not to seek care for symptoms of BV directly when symptoms appear. The mean time of beginning of symptoms was 11.3 months (median time 6 months) with a range of 1-60 months, prior to diagnosis. We have no explanation for this but if this is the same in all countries it could be a possible explanation why STI clinics have difficulties to find a possible connection between a new sexual partners and symptoms of BV. As 35 women reported that they had symptoms of BV after they started a new relationship it was possible to calculate an "incubation time". This "incubations time" is only speculative but nevertheless interesting, since it is only a median of 2 months.

## Conclusion

The study shows that aggressive treatment of the patient with antibiotics combined with *Lactobacillus *administration can provide a long lasting cure. A striking result of our study is that a change of partner is strongly associated with relapse of BV giving support for the notion that BV is an STD and that the patient's sexual partner needs to be treated. The new *Lactobacillus *preparation tested were as good as the commercial EcoVag^® ^product when administered intra vaginally but vaginal colonisation observed for some strains did not seem to influence the cure rate. Further studies with extended treatment with different preparations of lactobacilli and larger cohorts will have to be performed to evaluate the relationship between colonisation by lactobacilli and cure of BV.

## Competing interests

This study has been performed with the economical support from Bifodan AS. The company has had no influence on study design, data interpretation or content of the article.

## Authors' contributions

P-GL, UF and LH have contributed to the study design; P-G L, collection of clinical data and analysis of air-dried smears, KKA and HM to the characterisation of the lactobacilli; HM, UF and SP to the cultivation and typing of the lactobacilli and SN to statistical analysis. EB have performed the encapsulation of the freezed-dried lactobacilli. All have contributed to the writing of manuscript and all authors read and approved the final manuscript.

## Pre-publication history

The pre-publication history for this paper can be accessed here:

http://www.biomedcentral.com/1471-2334/11/223/prepub
